# Case series: Computed tomography (CT) demonstrates lacrimal canal involvement in koalas (*Phascolarctos cinereus*) with maxillary incisor dental disease

**DOI:** 10.1111/avj.13158

**Published:** 2022-03-25

**Authors:** AJ Bryce, ME Milne, D Tyrrell, K Bodley

**Affiliations:** ^1^ Department of Veterinary Clinical Sciences, Melbourne Veterinary School, Faculty of Veterinary and Agricultural Sciences University of Melbourne Melbourne Australia; ^2^ Melbourne Zoo, Veterinary Zoos Victoria Melbourne Australia

**Keywords:** computed tomography of koalas, koala dental disease, nasolacrimal duct

## Abstract

Dental disease is common in wild and captive koalas. Effective treatments are limited and dental disease may not be recognised until it is quite severe. We describe the appearance of varying severities of dental disease on computed tomography (CT) images in a case series of six koalas. This case series demonstrates the use of CT to detect both mild and severe dental disease in koalas. The study also documents the normal CT appearance of the nasolacrimal duct in the koala. The only clinical abnormality in koalas with the mildest dental disease was ocular discharge. Computed tomography findings associated with ocular discharge were periapical lysis of first maxillary incisors, lacrimal canal remodelling and subsequent nasolacrimal duct obstruction. Dental disease should be a differential diagnosis for ocular discharge in koalas and CT examination enables visualisation of early stages of dental disease.

## Introduction

Dental disease is common in wild and captive koalas (*Phascolarctos cinereus*) with a previous study reporting the prevalence of 86% and 50% respectively.^1^ Weight loss is a commonly reported sign of dental disease, but sub‐clinical disease is often detected on routine oral examination. Management of chronic dental disease remains challenging in this species; considering this, early detection of dental disease could help guide clinicians in establishing early intervention and appropriate management plans.

A variety of factors contribute to the development of dental disease in koalas.^1^, ^2^, ^3^, ^4^, ^5^ Dietary requirements result in continuous grinding of molar teeth; this progressive wear and erosion of crowns with age predisposes aged koalas to periodontal disease. Malocclusion also contributes to excessive wear of the occlusal surfaces and subsequent pulp exposure, allowing bacteria to ascend and cause periodontitis or endodontitis.^1^, ^2^ Periapical lysis can occur secondary to periodontitis from bacteria such as Porphyromonas sp.,^4^ and the role of retroviral infection modulation of the immune response to oral bacterial infections have also been explored.^2^ The location of most dental disease in koalas is usually the maxillary incisor teeth^2^; in a population of wild‐caught koalas the most common site of increased mobility was found in the maxillary incisor teeth.^2^

The clinical sign of serous ocular discharge in koalas is commonly attributed to *Chlamydia pneumoniae* and *C. pecorum* infections.^6^, ^7^ Potential causes of this sign are, however, more diverse; ocular discharge can occur secondary to inflammatory and infectious conjunctival conditions, excessive lacrimal gland secretions and obstruction of nasolacrimal duct secondary to dental disease. The latter has been reported in a cat^8^ and rabbits.^9^ Unilateral ocular discharge related to the dental disease is also documented in the kangaroo,^10^ but has yet to be reported in the koala.

The function of the nasolacrimal duct is to drain the tears from the eye and excrete them into the rostral nasal cavity.^11^ Similar to other species, in the koala the lacrimal punctae are positioned 2–3 mm from the medial canthus, with the terminal opening on the ventrolateral aspect of the nasal floor.^10^ In the koala the terminal portion of the nasolacrimal duct is separated by a thin portion of the incisive bone from the apex of the first maxillary incisor tooth.^12^ The intra‐nasal anatomy and course of the nasolacrimal duct has not been documented in the koala; a publication documenting the CT anatomy of the nasal cavity and paranasal sinuses of the koala^13^ did not include a description of the lacrimal canal and nasolacrimal duct. The adult koala has 30 teeth with the following dental formula^10^: 2 × Incisors $\frac{3}{1}\;$ Canine $\frac{1}{0}$ Premolar $\frac{1}{1}$ Molar $\frac{4}{4}$. Similar to domestic cats and dogs, koala teeth consist of a crown with an external enamel layer, an internal dentine layer and a pulp cavity.^14^ The root of the tooth has an external layer of cementum and more central dentine and pulp cavities.^14^ The root is embedded in alveolar bone and surrounded by a periodontal ligament.^14^

In this two‐part study, we use CT dacryocystography to document the normal anatomy of the nasolacrimal duct and relationship to dentition in the koala. The second part of the study is a retrospective case series aimed at demonstrating the utility of computed tomography (CT) to characterise the extent of dental disease in a sample of captive koalas. We hypothesise that ocular discharge is an early clinical sign of dental disease, and that CT would enable detection of lacrimal canal abnormalities secondary to dental disease.

## Materials and methods

### Part 1. Anatomy of the nasolacrimal duct in the koala

To document the normal pathway of the nasolacrimal duct, a positive contrast computed tomographic dacryocystogram was conducted on a mature free‐range koala undergoing necropsy for an unrelated reason. The cadaver had a grossly normal head, with no palpably loose teeth or evidence of dental disease such as tooth discoloration or fractures identified on visual inspection. A transverse plane CT was acquired using 16 slice CT scanner (Siemens Emotion, Siemens, Erlangen, Germany). A survey CT scan of the head was reconstructed for bone, 1.0 mm thick slices. The distal opening of the right nasolacrimal orifice was catheterised near the nares, using a 24G intravenous catheter, 2 mL of Iohexol 240 mg/mL (Omnipaque, GE Healthcare, Chicago, USA) was injected by hand, and acquisition was repeated. CT data were reconstructed as standard three plane multiplanar reformats, curved parasagittal multiplanar reformats to follow the course of the nasolacrimal duct, and a three‐dimensional model was developed. The course of the nasolacrimal duct and relationship to dental structures was described.

### Part 2. Retrospective case series

Koalas who had a CT examination of the head conducted at U‐Vet Werribee, Australia, were identified by searching the imaging database (Synapse, Fujifilm, Tokyo, Japan). Over the 14‐year duration of the imaging database, between July 2007 and November 2021, six koalas were identified. All were captive or free ranging animals referred from two local zoos. Patient demographic features, history including indication for CT examination of the head, and clinical findings were recorded. Imaging findings were recorded by review of the report generated at the time of imaging, and consensus review by a resident in veterinary radiology (AB) and a registered specialist in veterinary radiology (MM).

During the study period, one CT examination was conducted on a single slice CT scanner (X‐vision/GX, Toshiba, Kawasaki, Japan), with images only available as transverse plane 1 mm thickness bone window images. The remaining five CT examinations were performed on a 16 slice CT scanner (Siemens Emotion, Siemens, Erlangen, Germany), with both soft tissue and bone window images available in orthogonal multiplanar reformats with slice thickness of 1 to 3 mm.

Features of dental disease visible on CT were recorded as teeth that were normal, absent, if periapical lysis was present, if widespread alveolar bone lysis was present encompassing multiple teeth or tooth roots, if teeth were fractured, and if an oronasal fistula was present.

Lacrimal canals and nasolacrimal ducts were compared to the contralateral side to assess for normality, or if bilateral disease was suspected the study was compared to a study of a koala with a normal head, undergoing CT for reasons unrelated to the dental or ocular systems.

## Results

### Part 1. Anatomy of the nasolacrimal duct in the koala

**FIGURE 1:**
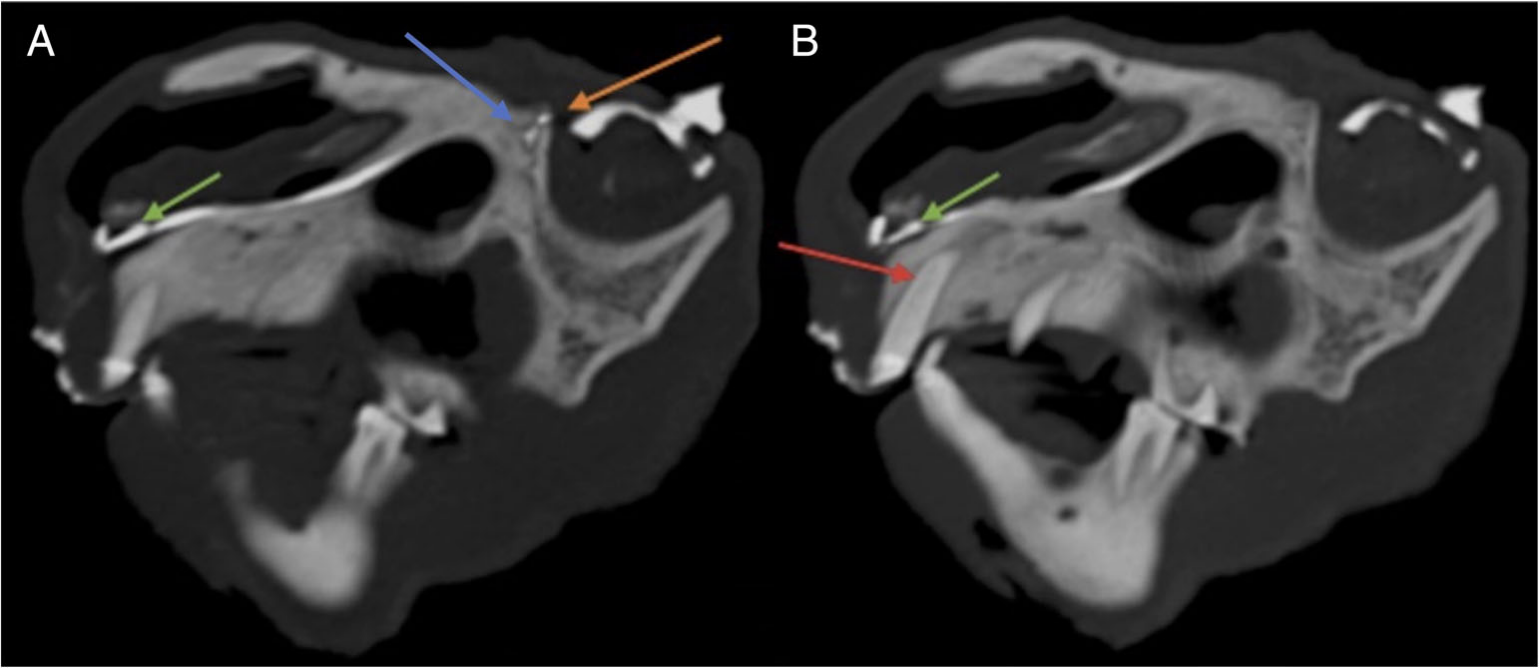
(A) CT dacryocystography: A curved sagittal maximum intensity projection multiplanar reformatted CT image showing the passage of the nasolacrimal duct. (B) the same CT series two slices axially, to demonstrate the proximity of the maxillary first incisor tooth root to the duct. The contrast filled nasolacrimal duct is shown between green and orange arrows in both images. The orange arrow depicts the dorsal lacrimal canaliculus and the blue arrow shows the nasolacrimal duct at the level of the caudal lacrimal foramina with union of the dorsal and ventral canaliculi (the entire ventral canaliculi is not shown in this image). The green arrow shows the terminal aspect of the nasolacrimal duct and the red arrow illustrates the left maxillary first incisor tooth. Artefactual contrast can be seen around the lips and orbits in both images

**FIGURE 2:**
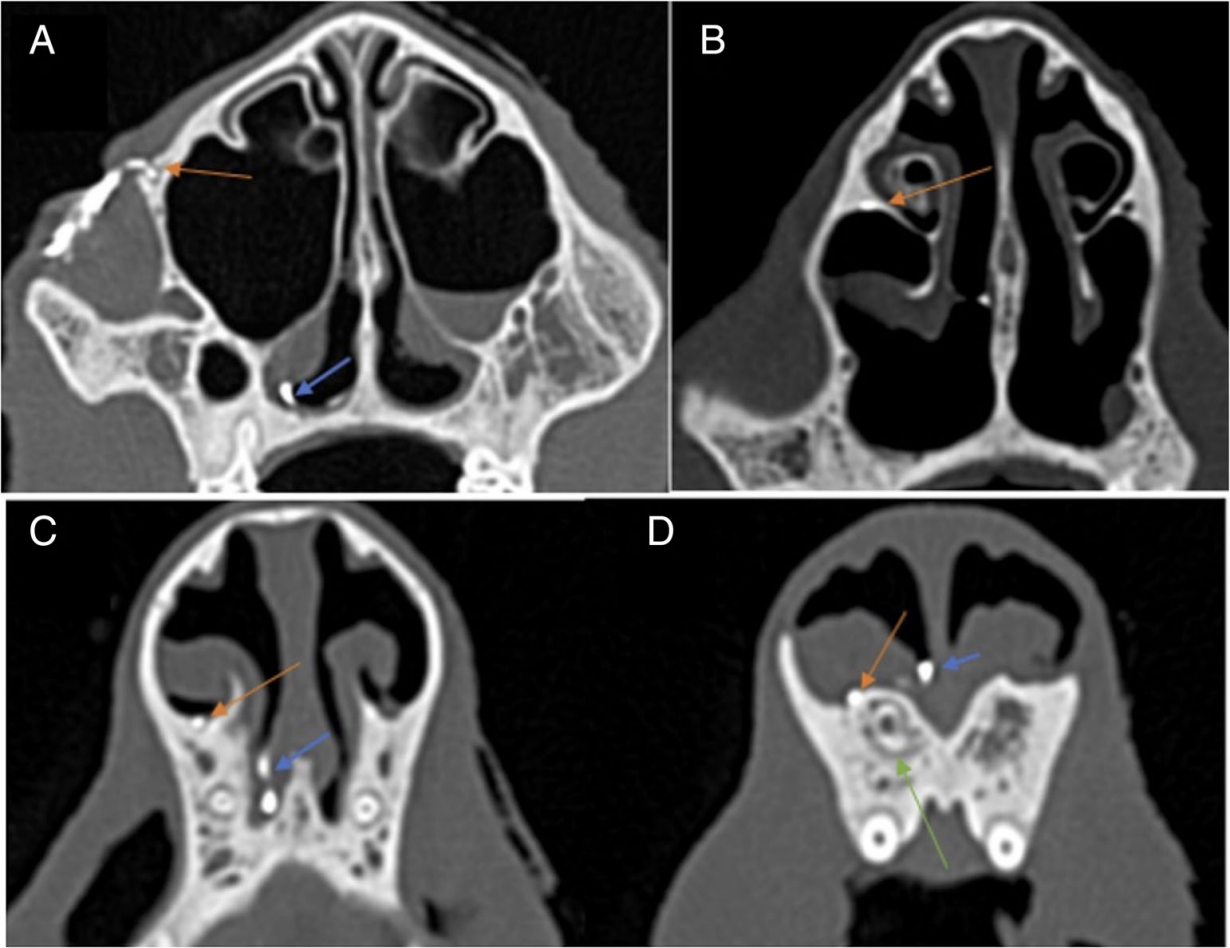
Serial transverse plane bone window images from caudal to rostral (A–D), after dacryocystography, showing the course of the lacrimal canal and nasolacrimal duct (orange arrows) the blue arrow demonstrates iatrogenic contrast leakage. Image (A) shows contrast leaking from the dorsal puncta (orange arrow) into the surrounding palpebral tissue. The green arrow in image (D) shows the apex of the first right maxillary incisor

The entire nasolacrimal duct was visualised with CT dacryocystogram (Figure 1). The duct wall had smooth margins and as is described in other mammalian species the duct could be divided into three portions: palpebral, osseous and membranous.^15^ The palpebral portion consisted of dorsal and ventral punctae positioned approximately 2 mm caudal to the medial canthus, both punctae had corresponding canaliculi that continued rostrally (Figure 2A). The dorsal and ventral canaliculi united at the caudal lacrimal foramina to form the intra‐osseous portion of the nasolacrimal duct (as shown in Figure 1). The duct continued rostro‐ventro‐axially in the osseous lacrimal canal through the lacrimal bone and maxilla (Figure 2B) to enter the nasal cavity through the rostral lacrimal foramina ventroabaxial to the ventral nasal concha (Figure 2C). The membranous portion of the nasolacrimal duct continued rostroaxially from the foramina and opened at the nasolacrimal orifice ventrally in the ventral nasal meatus at the level of the apex of the first maxillary incisor (Figure 2D). Upon injection of contrast for CT dacrycystogram, leakage was noted around the hub of the catheter and into the nasal cavity and after injection contrast was noted around the orbit. The contrast in the nasal cavity and around the orbit were deemed iatrogenic as no defects or fistulas were identified in the nasolacrimal duct.

The measurements of the duct size via dacryocystography were consistent with an anatomical study reference values:

1. The nasolacrimal duct length approximately 45.0 mm (anatomical reference of 28.0–50.0 mm).^16^
2. Nasolacrimal duct diameter (anatomical reference 0.3 mm −1.4 mm)^16^:

- Dorsal canaliculus – 0.8 mm in diameter.
- Osseous portion – 1.0 mm in diameter.
- Membranous portion – 1.1 mm in diameter.

### Part 2. Retrospective case series

Six koalas were identified who had a CT examination of the head during the study period. There were four captive male koalas, one captive female and one free‐ranging female koala. Koalas were aged between 3 and 8 years, with median age of 6.5 years. One koala, a 6‐year‐old captive female (Case 1) had a head CT acquired 21 months prior for an unrelated clinical problem. The head was normal at that earlier examination, and this normal study was used for comparison in the other cases.

Two koalas presented with right‐sided ocular discharge; one of these koalas also had blepharospasm. In both, ocular discharge was the main presenting complaint, without clinical evidence of underlying dental disease. In the other four koalas, dental disease was suspected or directly observed; one koala had a discoloured right maxillary incisor tooth, one had a history of dental‐associated osteomyelitis and presented with facial swelling responsive to antibiotics, one presented with weight loss and an oronasal fistula at the left maxillary first molar tooth, and the other had chronic weight loss without the observable dental disease. Details of signalment, presenting signs, and a summary of CT findings are provided in Table 1.

**TABLE 1:** Summary findings from six koalas with dental disease identified by head CT

Case number	Signalment	Presenting signs	CT abnormalities
1	6‐year old female captive	Unilateral, right epiphora and mild blepharospasm, 6‐day duration	Peri‐apical lysis right first maxillary incisor tooth. Distal expansion of right lacrimal canal, filled with soft tissue. (Figure 5)
2	3‐year old male captive	Unilateral right epiphora	Peri‐apical lysis right first maxillary incisor tooth. 4 mm defect right ventral nasal conchae, ventral to right lacrimal canal. Slight distal expansion of the right lacrimal canal. Dacryocystography: Partial obstruction of the right nasolacrimal duct.
3	3‐year old female free ranging	Discoloured right maxillary incisor tooth	Peri‐apical lysis right and left maxillary first incisor teeth, mild on the left side. Fractured root left maxillary first incisor tooth. Distal expansion of the lacrimal canals bilaterally. Soft tissue swelling caudodorsal to the nasal cavity on the right side.
4	8‐year old male captive	Chronic dental‐associated osteomyelitis. Right sided facial swelling, 5 days duration, responsive to antibiotics	Peri‐apical lysis left maxillary first incisor tooth with a fractured root. Absent right maxillary first, second and third incisor teeth, with extensive lysis of the incisive bone with oronasal fistula, and periosteal new bone formation around the incisive bone. Distal expansion of the lacrimal canals bilaterally, more severe on the right side.
5	7‐year old male captive	Oronasal fistula associated with left maxillary first molar tooth. Weight loss.	Peri‐apical lysis right and left maxillary first incisor teeth. Lysis extends abaxially to margin of the incisive bone. Distal expansion of the lacrimal canals. Absent left maxillary first molar tooth with oronasal fistula. Peri‐apical lysis left maxillary third and fourth molar teeth with diastema, absent left mandibular first molar tooth.
6	7‐year old male captive	Chronic weight loss	Peri‐apical lysis right and left maxillary first incisor teeth, extending abaxially to lateral margins of the left incisive bone with a 3 mm defect in the lateral margin of that bone. Periosteal new bone right incisive bone. Distal expansion of the left lacrimal canal. (Figure 4)

**FIGURE 3:**
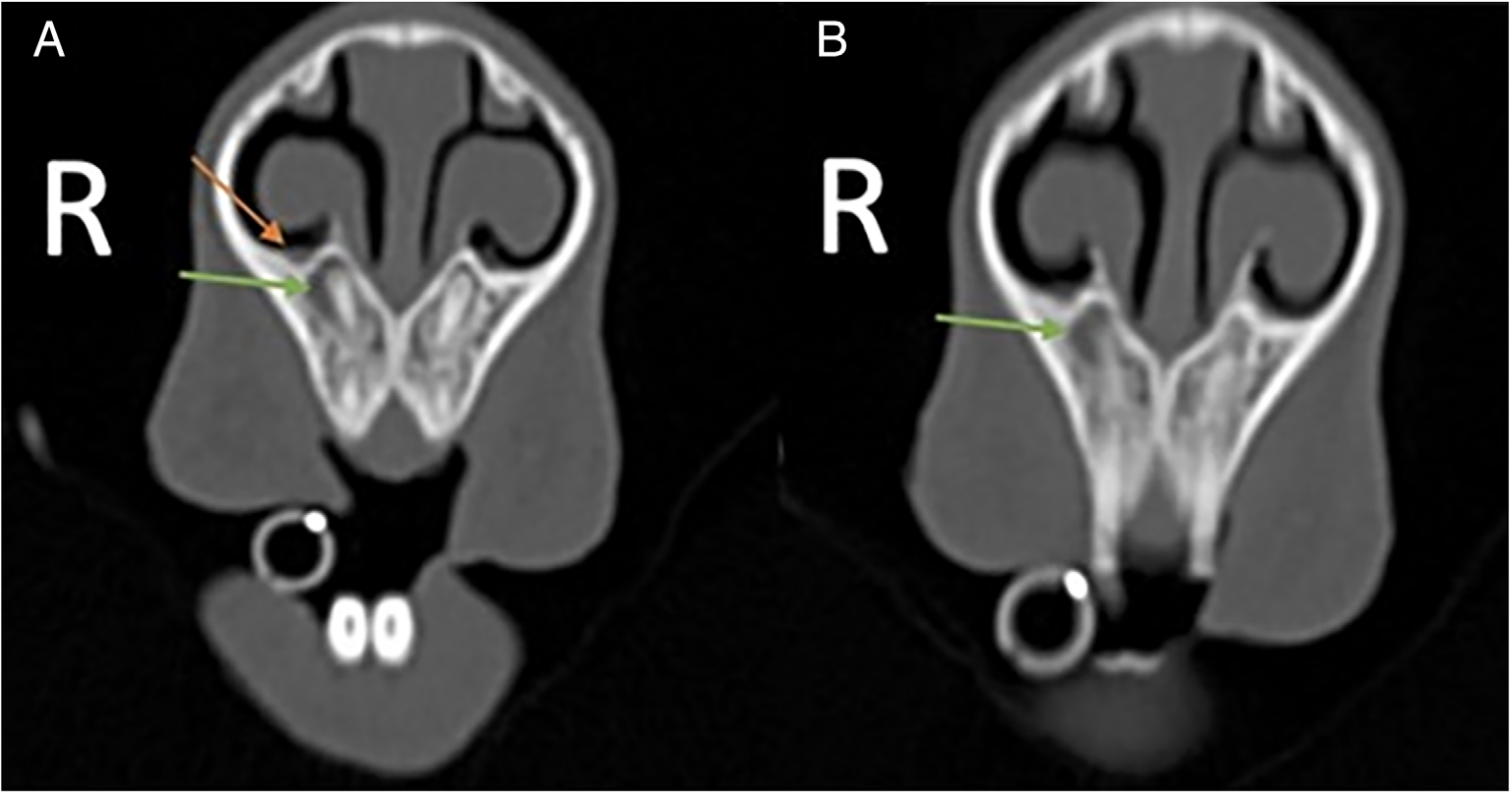
Case 1. Transverse images at the level of the apex of the first maxillary incisor made at (A) 1 mm slice thickness compared with (B) 3 mm slice thickness. There is periapical lysis associated with the first right maxillary incisor (green arrow), the visualisation of the distended right nasolacrimal orifice (orange arrow) is superior in image (A) compared to (B)

In all koalas, peri‐apical bone lysis was observed in one or both first maxillary incisor teeth. In all koalas, there was alteration to one or both lacrimal canals, which comprised focal expansion of the distal portion of the lacrimal canal to varying extents with filling of the canal with soft tissue, and soft tissue swelling at the distal nasolacrimal puncta. One koala (Case 1) had transverse plane bone window images reconstructed with 3, 2 and 1 mm slice thickness; soft tissue change at the distal nasolacrimal puncta were difficult to appreciate with the 3 mm thick slices, but readily seen on 1 mm thick slices. (Figure 3).

A koala with right epiphora and normal dental examination (Case 2) had peri‐apical lysis of the right maxillary first incisor tooth, with soft tissue swelling at the distal nasolacrimal puncta; this was catheterised and dacryocystography was performed, which confirmed partial obstruction of the duct by soft tissue.

One koala (Case 4) presented with chronic osteomyelitis and facial swelling. The right first, second and third maxillary incisor teeth were all absent with a large area of lysis of the incisive bone, peri‐apical lysis and a fractured root of the left maxillary first incisor tooth, and bilateral dilation of the lacrimal canals, worse on the right side.

Periodontal disease of the cheek teeth was only identified in one koala (Case 5), a 7‐year old male with poor body condition. In addition to peri‐apical lysis of right and left maxillary first incisor teeth, that koala had an absent left maxillary first molar tooth with an oronasal fistula, peri‐apical lysis of the left maxillary third and fourth molar teeth with a diastema, and an absent left mandibular first molar tooth.

## Discussion

Computed tomography allowed visualisation of nasolacrimal duct disease secondary to dental disease in every koala in this case series. The koala nasolacrimal anatomy could be easily documented after CT dacrycystogram. Similar to rabbits, the terminal portion of the Koala nasolacrimal duct and orifice are in close proximity to the root of the first maxillary incisor.^15^, ^17^ This proximity most likely accounts for the prevalence of nasolacrimal duct changes seen with maxillary incisor dental disease in this study. Contrasting to other domestic species the Koala lacks a lacrimal sac, reducing their capacity to store lacrimal secretions, this is supported by a previous anatomical study.^15^, ^16^

**FIGURE 4:**
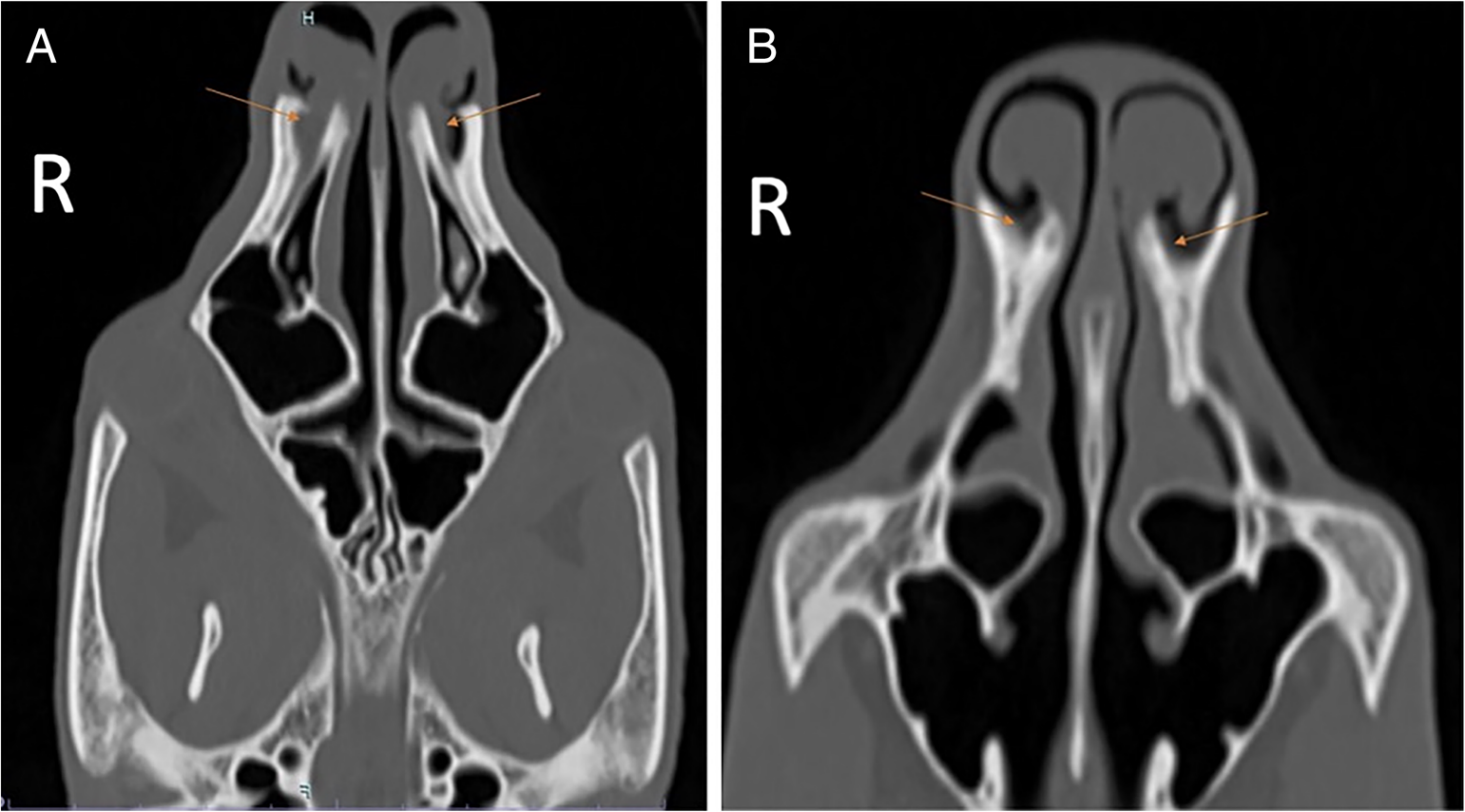
Image (A) is a dorsal reformat in a bone window of Case 6, a 7 year old captive male koala with chronic weight loss. The nasolacrimal duct is indicated by orange arrows. There is expansion of the distal aspect of the right lacrimal canal which is filled with soft tissue. Similar but less severe expansion is noted on the left side. (B) Is a CT of Case 1 made at a time when the patient was asymptomatic for dental disease or epiphora, and is regarded as normal. The orange arrows show the distal aspects of the nasolacrimal ducts, which are symmetric and small in size

**FIGURE 5:**
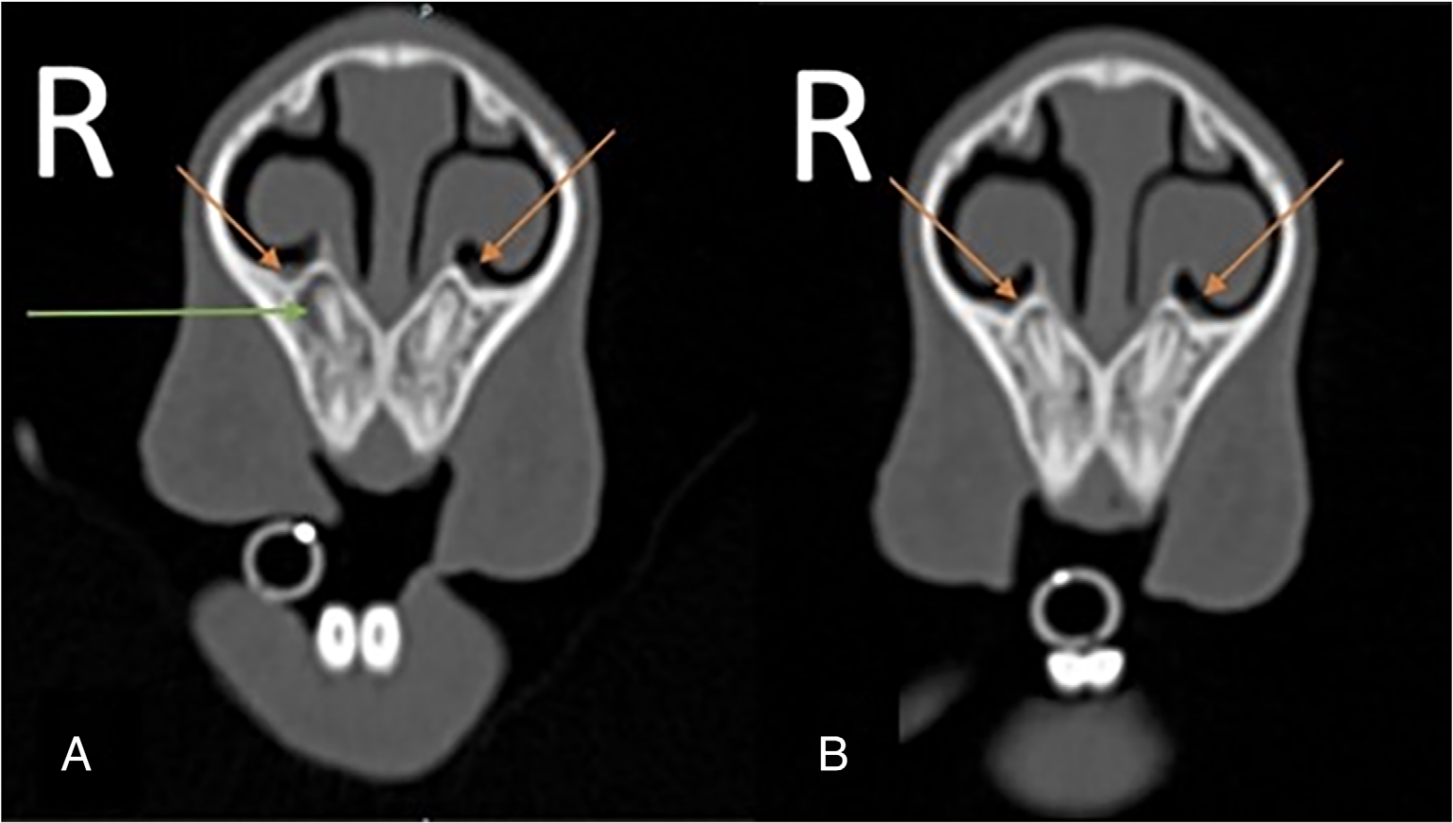
Transverse images in a bone window of Case 1 made at the time of presentation for right sided epiphora (A) and made 21 months earlier, for an unrelated problem (B). Both images are at the level of the apex of the first maxillary incisors. Image (A) 1 mm slice thickness showing peri‐apical alveolar bone lysis indicated by the green arrow and the nasolacrimal ducts by the orange arrows. The terminal right nasolacrimal duct is enlarged compared to the contralateral side and the previous image shown in image B. Image (B) 1 mm slice thickness at the same location no CT abnormalities identified. Note the lack of periapical alveolar bone lysis on image (B)

In our case series koalas presenting with clinical signs such as facial swelling and weight loss or poor body condition had more extensive periodontal disease and lacrimal canal changes. Cases 4, 5 and 6 (Figure 4) had extensive alveolar bone loss which extended beyond the periapical region of the first maxillary incisors. In Case 4, osteolysis continued latero‐ventrally and an osseous deficit was seen on the abaxial surface of the incisive bone associated with an irregular periosteal reaction consistent with extensive osteomyelitis. The two koalas presenting solely with epiphora had the least extensive osseous changes associated with dental disease, confined to the periapical region of the first maxillary incisor with localised effect on the nasolacrimal duct. In case 1 a dental examination was conducted prior to CT and no abnormalities were noted. The CT and dental examination findings of Case 1 suggest that when dental disease is confined to the periapical region of the first maxillary incisors (the most commonly affected teeth in koalas), ocular discharge may precede other clinical and dental examination findings (Figure 5). This is most likely the result of the proximity of the nasolacrimal duct to the roots of the first maxillary incisor teeth, and tortuous nature of the lacrimal canal in this species.

Although dental radiographs can be used to diagnose periapical lysis associated with dental disease, CT has the advantage of being able to reformat images in multiple planes and eliminate the superimposition of structures. Computed tomography also has superior contrast resolution for soft tissue compared to radiographs. Both factors contribute to the superior ability of CT compared to dental radiographs to visualise periodontal disease and the nasolacrimal ducts in the koala. The use of CT‐dacryocystography is also a useful technique to document patency or occlusion of the nasolacrimal duct.

The availability of transverse images at different slice thicknesses as seen in Case 1 highlights the importance of reconstructing CT data with as thin slices as possible, as subtle lesions were not clearly visible on 3 mm thick reformats. The entire length of the nasolacrimal duct could also only be confidently traced on the 1 mm thick slices. The termination of the nasolacrimal duct could be visualised on the 2 mm thick slices but it was difficult to trace the entire duct. The authors recommend 1 mm thick reconstructions or thinner to permit complete assessment of the nasolacrimal duct and the apex of the first maxillary incisors in the koala.

This study has several inherent limitations due to its retrospective nature. The tooth wear class was not available on review of the koala's history and some histories of more chronic cases were incomplete, lacking information on the incipient signs of disease. The normal CT dimension of the koala nasolacrimal duct is not published and so an interpretation of dilation or obstruction is based on comparison with the contralateral duct or a presumed normal animal. The study also lacks histopathologic verification of the CT diagnosed nasolacrimal duct pathology.

This study illustrates the use of CT as a valuable diagnostic tool for the examination of nasolacrimal and dental pathology in koalas. In this case series, we found CT was able to detect nasolacrimal duct obstruction secondary to maxillary incisor dental disease, even in cases with occult dental disease. Epiphora is a readily detectable clinical sign which can be seen with early dental disease. Early detection is key to further our understandings of dental disease in koalas and developing effective treatments. Therefore, it is proposed that koalas with ocular discharge or epiphora of an unknown aetiology undergo a CT examination to rule out maxillary incisor dental disease.

## Conflict of interest and sources of funding

The authors declares no financial or personal conflict of interests.

## References

[1] Pettett L . Oral health in south East Queensland koalas: Prevalence of periodontal disease and other pathologies. St Lucia, University of Queensland, 2016.

[2] Butcher R , Pettett L , Fabijan J et al. Periodontal disease in free‐ranging koalas (Phascolarctos cinereus) from the Mount Lofty Ranges, South Australia, and its association with koala retrovirus infection. Australian Vet J 2020;98:200–206. 31971256 10.1111/avj.12919

[3] Lee EF , Varanasi S , Pettett LM et al. Loss of tooth‐supporting bone in the koala (Phascolarctos cinereus) with age. Australian J Zoo 2011;59:49–53.

[4] Bird PS , Huynh SC , Davis D et al. Oral disease in animals: the Australian perspective. Isolation and characterisation of black‐pigmented bacteria from the oral cavity of marsupials. Anaerobe 2002;8:79–87.

[5] DeSantis LR , Alexander J , Biedron EM et al. Effects of climate on dental mesowear of extant koalas and two broadly distributed kangaroos throughout their geographic range. PLoS one 2018;13:e0201962. 30133503 10.1371/journal.pone.0201962 PMC6104949

[6] Cockram F , Jackson A . Keratoconjunctivitis of the koala, Phascolarctos cinereus, caused by Chlamydia psittaci. J Wildlife Dis 1981;17:497–504. 10.7589/0090-3558-17.4.497 7338971

[7] Jackson M , White N , Giffard P et al. Epizootiology of Chlamydia infections in two free‐range koala populations. Vet Microbiol 1999;65:255–264. 10223324 10.1016/s0378-1135(98)00302-2

[8] Anthony JMG , Sandmeyer LS , Laycock AR . Nasolacrimal obstruction caused by root abscess of the upper canine in a cat. Vet Ophthalmol 2010;13:106–109. 20447029 10.1111/j.1463-5224.2009.00754.x

[9] Florin M , Rusanen E , Haessig M et al. Clinical presentation, treatment, and outcome of dacryocystitis in rabbits: a retrospective study of 28 cases (2003–2007). Vet Ophthalmol 2009;12:350–356. 19883464 10.1111/j.1463-5224.2009.00727.x

[10] Vogelnest L , Woods R . Medicine of Australian mammals. Csiro Publish 2008;1:227–328.

[11] Noller C , Henninger W , Gronemeyer DH et al. Computed tomography‐anatomy of the normal feline nasolacrimal drainage system. Vet Radiol Ultrasound 2006;47:53–60. 16429985 10.1111/j.1740-8261.2005.00105.x

[12] Kratzing JE . The anatomy and histology of the nasal cavity of the koala (Phascolarctos cinereus). J Anat 1984;138(Pt 1):55–65. 6706839 PMC1164310

[13] Hemsley S , Palmer H , Canfield R et al. Computed tomographic anatomy of the nasal cavity, paranasal sinuses and tympanic cavity of the koala. Australian Vet J 2013;91:353–365. 23980827 10.1111/avj.12098

[14] Pettett LM , McKinnon AJ , Wilson GJ et al. The development of an oral health charting system for koalas (Phascolarctos cinereus). J Vet Dent 2012;29:232–241. 23505786 10.1177/089875641202900404

[15] Ali MJ , Rehorek SJ , Paulsen F . A major review on disorders of the animal lacrimal drainage systems: evolutionary perspectives and comparisons with humans. Ann Anat Anatomischer Anzeiger 2019;224:102–112. 31071433 10.1016/j.aanat.2019.04.003

[16] Kempster R , Bancroft B , Hirst L . Intraorbital anatomy of the koala (Phascolarctos cinereus). Anatom Rec Off Publice Am Assoc Anatom 2002;267:277–287. 10.1002/ar.10118 12124905

[17] Fritz J , Gaillot H , Ruel Y . Helical computed tomographic‐dacryocystography in adult pet dwarf rabbits: procedure and normal appearance. Vlaams Diergeneeskundig Tijdschrift 2020;89:299–308.

